# The effect of COVID-19 on the home behaviours of people affected by dementia

**DOI:** 10.1038/s41746-022-00697-4

**Published:** 2022-10-17

**Authors:** Alina-Irina Serban, Eyal Soreq, Payam Barnaghi, Sarah Daniels, Rafael A. Calvo, CR&T Group, David J. Sharp

**Affiliations:** 1 UK Dementia Research Institute, Care Research and Technology Centre, London, UK; 2 Dyson School of Design Engineering, Imperial College London, London, UK; 3 Department of Brain Sciences, Imperial College London, London, UK

**Keywords:** Predictive markers, Dementia, Geriatrics, Occupational health

## Abstract

The COVID-19 pandemic has dramatically altered the behaviour of most of the world’s population, particularly affecting the elderly, including people living with dementia (PLwD). Here we use remote home monitoring technology deployed into 31 homes of PLwD living in the UK to investigate the effects of COVID-19 on behaviour within the home, including social isolation. The home activity was monitored continuously using unobtrusive sensors for 498 days from 1 December 2019 to 12 April 2021. This period included six distinct pandemic phases with differing public health measures, including three periods of home ‘lockdown’. Linear mixed-effects modelling is used to examine changes in the home activity of PLwD who lived alone or with others. An algorithm is developed to quantify time spent outside the home. Increased home activity is observed from very early in the pandemic, with a significant decrease in the time spent outside produced by the first lockdown. The study demonstrates the effects of COVID-19 lockdown on home behaviours in PLwD and shows how unobtrusive home monitoring can be used to track behaviours relevant to social isolation.

## Introduction

The COVID-19 pandemic has dramatically altered the behaviour and daily activities of most of the world’s population. In the UK, people living with dementia (PLwD) have been particularly affected^1,2^. Dementia is the most common co-morbidity associated with COVID-19 deaths in the first wave of the pandemic^3^, and public health measures aimed at slowing virus spread have dramatically affected PLwD through widespread social distancing and home ‘lockdown’ measures^2,4^. These measures have protected individuals from infection, while dramatically reducing social interactions and raising concerns about the detrimental effects of social isolation^5,6^. Hence, there is a need to directly assess the impact of public health measures in vulnerable populations, both to investigate how well individuals with cognitive impairment can follow public health advice and to measure the social impact of these measures.

The use of connected sensing technologies enables continuous remote monitoring of the health and well-being of people at risk of social isolation^7^. Wearable devices provide direct information about physical activity^8^ but are challenging for PLwD because of problems with compliance related to their cognitive impairments^9^. Passive sensor networks provide an attractive alternative as they allow unobtrusive and continuous monitoring of home activity without the need for user engagement^10^. We report data from an ongoing study of dementia care that uses environmental sensors deployed in the home of PLwD, including passive infra-red sensors, door sensors and smart plugs. Continuously collected information about home daily behaviour through the recent pandemic has provided insights into how COVID-19 has affected the lives of PLwD.

The first national UK lockdown started on 23 March 2020 and introduced social distancing, self-isolation and limited time for outdoor activities. These measures were relaxed mid-May 2020; however, new restrictions were announced to aid in further reducing the spread of the pandemic. Here we present a quantitative analysis of activity levels from the homes of PLwD through the COVID-19 pandemic. Sensor data from the cohort of households were collected before and during the UK COVID-19 lockdowns and were used for continuous assessment of the daily activities. We investigate overall home activity levels and the duration of time spent outside calculated using a custom algorithm, described below in the methods section. This algorithm is developed for this study to determine when the occupants of the house are outside by combining door use with in-home activity levels. The effects of single and multiple occupancies are also explored, as PLwD living alone is more likely to experience social isolation^11^. The analysis provides insights into how household activity and time spent outside have changed through the pandemic and how PLwD adapted to the changing pandemic restrictions.

## Results

**Fig. 1:**
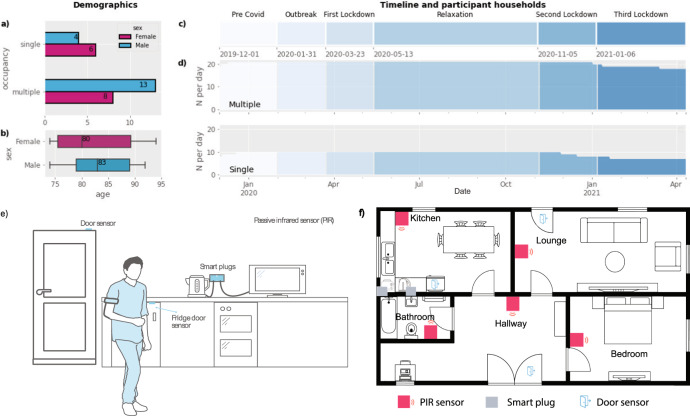
Cohort description and home monitoring system. **a**, **b** Household occupancy, sex and age distributions (boxplot shows the minima and maxima values, lower and upper quartiles, and the medians); **c** Timeline for data collection over the COVID-19 pandemic; **d** Number of participant households throughout the study; **e** Sensors contributing to the data reported in this study: passive infra-red movement sensors on the wall and door, as well as smart plugs to monitor household appliance use; **f** typical layout of sensors in exemplar home.

Data was acquired from the homes of 31 PLwD between 1 December 2019 and 12 April 2021 using passive infra-red sensors, door sensors and smart plugs in five different rooms, two entry points and three kitchen appliances (Fig. 1c–f). In total, almost 7 million unique observations across 498 unique days were collected. Behavioural changes caused by the pandemic were examined over six time periods: P1 baseline activity level prior to the pandemic (1 December 2019–31 January 2020); P2 onset of COVID-19 pandemic before any measures being announced by the government (31 January 2020–23 March 2020); P3 introduction of lockdown measures such as social distancing, self-isolation and limited time for outdoor activities (23 March 2020–13 May 2020); P4 relaxation of measures and introduction of new restrictions focused on local lockdowns and working from home (13 May 2020–5 November 2020); P5 second lockdown and Tier 4 level restrictions (5 November 2020–6 January 2021); and P6 third lockdown (6 January 2021–12 April 2021).

### Higher daytime home activity levels during the pandemic

**Fig. 2:**
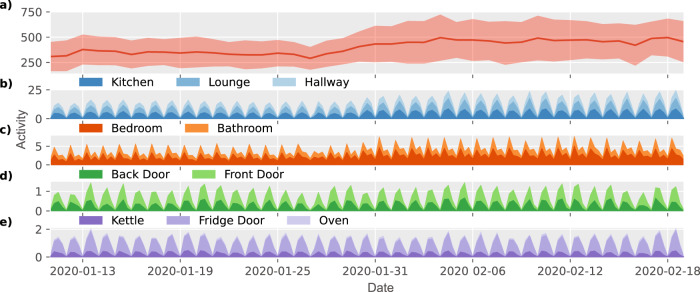
Change in home activity at the transition between P1 (pre-COVID-19 baseline) and P2 (COVID-19 outbreak). **a** Total mean daily activity summed across households from 10 January 2020 to 20 February 2020 demonstrating gradual increase in activity during the initial outbreak period (light pink represents standard deviation per day); **b**–**e** Mean activity summed across the different sensors and locations sampled at a 6 h frequency demonstrates the daily behavioural cycle across different sensors as well as the gradual increase in activity as a function of covid.

**Fig. 3:**
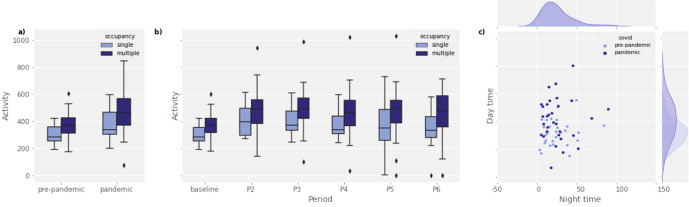
Changes in mean daily activity (sensor triggers) level during COVID-19. **a** Mean daily activity across households according to occupancy before and during COVID-19 (boxplot shows the minima and maxima values, lower and upper quartiles, and the medians); **b** Mean daily activity across households according to occupancy during the different pandemic periods (boxplot shows the minima and maxima values, lower and upper quartiles, and the medians) and **c** Distribution of mean daily activity across households during day and night before and during COVID-19.

The mean household activity was calculated based on the sum of daily activity across the different house sensors. Linear mixed-effects modelling (LME) and ANOVA were used to compare the baseline period (P1) to all other pandemic periods combined (P2-P6). The onset of the COVID-19 pandemic was associated with a significant increase in activity within the home, with a 14% change in total activity (LME ANOVA–F_(1, 31)_ = 30.05***, Figs. 2a, 3a, b). This increase is also observed in the mean activity summed across the different locations resampled at 6-h periods between 10 January and 20 February 2020. This demonstrates the gradual change in home activity as a function of the COVID-19 outbreak (Fig. 2b–e).

The main effect of overall changes in activity due to COVID-19 was seen in households with single and multiple occupancies (LME ANOVA–F_(1, 31)_ = 24.25***, Fig. 3a, Eq. (1)) with no interaction between occupancy and activity levels. The change in home activity between P1 and P2–6 was specific to the time of day (Fig. 3c, Eq. (2)). Activity levels changed between P1 and P2–6 during the day (05:00–23:00), but not during the night (23:00–05:00), with a significant interaction between the pandemic period (P1 and P2–6) and time of day (daytime/nighttime) (LME ANOVA–F_(1, 93)_ = 11.37***). A similar result was observed when the pandemic periods were modelled separately (Fig. 3b, Eq. (3)), with the main effect of activity change across the periods showing an overall increase from P1 onwards (LME ANOVA–F_(5, 152.17)_ = 6.97***) with no significant effect of occupancy (LME ANOVA–F_(1, 31.12)_ = 2.99, *p* = 0.09) and no interaction between the mean activity across periods and occupancy.

1 $${\mathrm{lmm}}\, <\!\!\! - {\mathrm{lmer}}({\mathrm{total}}\,{\mathrm{activity}}\sim {\mathrm{pandemic}} \ast {\mathrm{occupancy}} + (1|{\mathrm{subject}}))$$

2 $${\mathrm{lmm}}\, <\!\!\! - {\mathrm{lmer}}({\mathrm{total}}\,{\mathrm{activity}}\sim {\mathrm{pandemic}} \ast {\mathrm{daytime}} + (1|{\mathrm{subject}}))$$

3 $${\mathrm{lmm}}\, <\!\!\! - {\mathrm{lmer}}({\mathrm{total}}\,{\mathrm{activity}}\sim {\mathrm{pandemic}} \ast {\mathrm{period}} + (1|{\mathrm{subject}}))$$

### Relative changes in entryway events and time spent outside

**Fig. 4:**
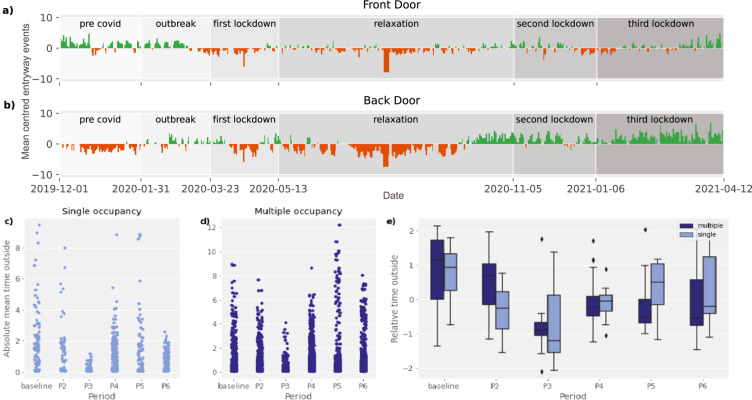
Changes in entryway events and time spent outside during COVID-19. **a**, **b** Mean-centred daily mean entryway events across the different periods for the front and back door; **c**, **d** Raw total daily time spent outside for single and multiple occupancy households for the six pandemic periods; **e** Standardised mean time spent outside across households during the different periods identified (boxplot shows the minima and maxima values, lower and upper quartiles, and the medians).

We next investigated how the pandemic affected the entryway events and the time individuals spent outside their homes. We calculated the daily mean of entryway events and mean-centred this across the periods. This demonstrated an overall reduction in door activity during the first lockdown and the relaxation period, followed by a relative increase in back door usage which suggests more garden activity (Fig. 4a, b). We quantified the time spent outside by identifying door usage (opening and closing) followed by the absence of in-home activity. A standardised measure of mean time spent outside per period per subject was used to allow comparison across the subjects. We examined the average time spent outside for each period while controlling for individual households and the number of household occupants using LME (Eq. (4)). Time spent outside was significantly different across the study periods (LME ANOVA–F_(5, 172)_ = 7.91***, Fig. 4c, d). As expected, all the pandemic periods exhibited reduced time spent outside relative to period 1. However, the first lockdown (see P3 in Fig. 4e) was associated with the most pronounced reductions (LME - t_(172)_ = −6.454***). The average time spent outside fell from a baseline average of 55 to 26 min.

4 $${\mathrm{lmm}} < - {\mathrm{lmer}}({\mathrm{relative}}\,{\mathrm{time}}\,{\mathrm{outside}}\sim {\mathrm{period}} \ast {\mathrm{occupancy}} + (1|{\mathrm{subject}}))$$

## Discussion

We used passive home monitoring of activity to investigate the effect of the COVID-19 pandemic on patients living with dementia (PLwD). In total, almost 7 million unique observations across almost 500 days were collected, providing a unique opportunity to study the effects of unique public health measures in this vulnerable population. Activity levels within the home increased across households early in the course of the pandemic. This change followed the World Health Organisation (WHO) declaration of a coronavirus outbreak but preceded the UK lockdown imposed by the UK government^12^, which suggests that people affected by dementia proactively changed their behaviour prior to public health restrictions imposed by the government.

Once imposed, UK lockdown restrictions limited movements outside the home, particularly in the first lockdown period (P3). We were able to directly study the impact of these public health measures on behaviour in the home. By recording front door usage and combining this with the measurement of subsequent activity in the home, we were able to derive a measure of time spent outside the home. This showed that lockdown restrictions produced a dramatic reduction in the time our cohort spent outside their home, which was particularly seen during the first UK lockdown period (P3: March–May 2020).

Investigating the effects of these unprecedented public health measures on vulnerable populations is important for several reasons. It allows investigation of how well vulnerable groups can follow public health advice and provides a way to measure its effects on household activity and social interactions. The strong correspondence between changes in household activity mandated by lockdown restrictions and actual household activity illustrates how well this vulnerable population complied with public health measures. Future work might extend this approach to both assess compliance with public health measures, and also the impact of these measures in vulnerable groups who might be at risk of unintended consequences such as social isolation.

In addition, our results show that remote sensor technology can be used to effectively monitor home behaviour and provides information about potential social care concerns within this population. The analysis provides information that is relevant to the assessment of social isolation in an unobtrusive automated way. For example, alerts about unexpected changes in time spent outside, either reductions or increases produced by wandering, could be responded to by social care teams or carers who are remotely providing support. This approach could assist in identifying unexpected changes in household activity, particularly the time spent outside the home. This is an important marker of day-to-day behaviour, which can be difficult to keep track of in vulnerable individuals. This information might be used as a digital biomarker to assist in targeting support provided by social care and primary healthcare to vulnerable individuals, such as those living with dementia.

Large reductions in the time individuals spend outside the home could have adverse effects on PLwD because increased social isolation is detrimental to the mental health of the elderly^5,6^. Neuropsychiatric problems such as anxiety and agitation can increase in patients with dementia who have fewer social interactions^13^, which is also associated with reductions in memory and functional abilities^14,15^. Confining individuals to their homes can also speed physical and cognitive decline in PLwD and increase caregiver burden^16,17^. In addition, health and social care have become more difficult to provide during periods of lockdown, further reducing the opportunity for social interactions. Hence, monitoring home activity levels provides information relevant to the neurological and psychiatric health of PLwD, and could be used to identify patients at risk of social isolation who would benefit from targeted support from care teams.

Our study has a number of potential limitations. One potential concern is that our participants behaviour may be impacted by the presence of sensors in their homes. We believe this is unlikely for a number of reasons. Our participants are part of an ongoing study conducted by the UK Dementia Research Institute, which investigates the use of technology to support people living with dementia with the main aim to develop new ways to identify health problems, such as falls and infections that lead to hospital admissions. This study started in April 2019, with participants recruited steadily since then. Moreover, the data reported here is collected from participants who have been part of the study for at least 6 months prior to the pandemic, hence, from the participants’ perspective, non-obtrusive sensor technology was unrelated to COVID-19. Also, at no stage was there any discussion about the participants’ behaviour in relation to compliance with COVID-19 protocols. This study provides an ideal measure of the effects of COVID-19 measures that occurred unexpectedly during the ongoing data collection.

The sensors deployed in this study provide specific information about moving around the house and the use of household appliances. Hence, it is not possible to use this approach to infer more detailed information about other aspects of cognitive function or detailed behaviours. This does not affect the results we report, but future research would benefit from deploying additional sensors capable of providing other types of information. Along similar lines, our set-up did not provide complete household coverage by sensors. The standard set-up for motion detection uses five passive infra-red sensors placed in the main rooms of the house, as identified by the participants, however, some participants can have more rooms than those with sensors. This creates challenges in understanding the behaviours that make use of those locations.

The sensor technology deployed allows us to monitor behaviour related to movements through the home. Although this does not provide detailed information about the cognitive or psychiatric state of an individual, the impact of large changes such as lockdown instructions can be identified, as we have demonstrated.

In summary, our results confirm the potential for passive monitoring of activity levels in the home of vulnerable adults. We show that unobtrusive home monitoring using passive infra-red (PIR) sensing technology can be used to measure home activity, derive indicators of social activity such as time spent outside the home and track the response of vulnerable populations to public health interventions. These digital measures have the potential to be developed as real-world digital biomarkers with utility in health and social care. Future work should focus on how best to incorporate these measures into the health and social care for vulnerable adults.

## Methods

### Study sample

Motion sensor data were collected during the COVID-19 pandemic from 31 households that formed part of an ongoing study of dementia conducted by the UK Dementia Research Institute (DRI), Care Research & Technology Centre. Data were continuously collected across a 16-month period inclusive of the three UK national lockdowns. Participants had a diagnosis of dementia or mild cognitive impairment, were more than 50 years of age (age range 75–93 years old, Fig. 1b), were living in the community and had a study partner who either lived with them or who was involved in their care. Full inclusion/exclusion criteria for the study are attached in the supplementary file. The study was ethically approved by the Surrey Borders Research Ethics Committee, and all participants provided written informed consent. Individuals were living alone in 10 households (4 male and 6 female). The other 21 households had more than one occupant (14 male and 7 female) (Fig. 1a).

### In-home monitoring technology

The households in this study had a range of Internet of Things (IoT) sensor technologies that were deployed in the home by Howz. The analysis reported uses anonymised binary data collected by Develco sensors (https://www.develcoproducts.com/) from households. Passive infra-red sensors (Develco Motion Sensor Mini) were installed in the bedroom, the lounge/living room, the kitchen, the bathroom and the hallway (Fig. 1e, f). Sensor placement varies according to the house layout, such that the sensors are placed within the most active locations within the house. Door sensors (Develco Window Sensor) were placed on the front door, the back door and the fridge door, and they collect information about a door being open or closed. Two smart plugs (Develco Smart Plug Mini) were placed on kitchen appliances such as the kettle and toaster or microwave, allowing the collection of data about the usage of these appliances.

As shown in our study, the sensors are capable of collecting movement data over long periods that we can interpret with the help of aggregate measures of household activity. The sensors used in the study are commercially available. Passive infra-red sensors are standard motion sensors that record a binary signal when motion is detected within a range of up to 9 m in a 45° up/down, left/right angle. Once a signal is recorded, there is a customisable off-time until the next trigger. The door sensor uses a magnetic sensor which detects and records two signals—the opening and closing of the door. The smart plug only records information about whether an appliance is in use or not. The accuracy of the data recorded can be affected by a number of technical factors, sensitivity and off-time, which are dependent on the customisation and placement of the sensors in the house. In our study, we obtain maximum sensitivity at around 3 m and have set the ‘off-time’ to 30 s. Furthermore, our system includes a monitoring team who responds both to clinical events and also technical failures. Data are monitored in near real-time as it enters the system, and sensor failures that might result from damage are identified and responded to by a maintenance team responsible for the deployment of the system into the home.

### Data acquisition

The analysis comprises of six stages that define the progression of the pandemic and the measures decided by the government. These stages are represented as periods in response to government announcements of the pandemic measures. A pre-COVID baseline is calculated from the data collected before the appearance of COVID-19 and that of any self-isolation measures being in place (P1). The analysis comprises of six stages that define the progression of the pandemic and the measures decided by the government. These stages are represented as periods in response to government announcements of the pandemic measures. Period two (P2) defines the time between the first case being recorded in the UK to the first lockdown. There are three national lockdown periods denoted by P3, P5 and P6. P4 denotes the relaxation period between the first and the second lockdown. The timeline is as follows (Fig. 1c):

- P1: pre-COVID-19 baseline (T0—1 December 2019 to T1—31 January 2020, 61 days)
- P2: COVID-19 outbreak (T1—31 January 2020 to T2—23 March 2020, 52 days)
- P3: first national lockdown (T2—23 March 2020 to T3—13 May 2020, 51 days)
- P4: relaxation (T3—13 May 2020 to T4—5 November 2020, 176 days)
- P5: second national lockdown (T4—5 November 2020 to T5—6 January 2021, 62 days)
- P6: third national lockdown (T6—6 January 2021 to T7—12 April 2021, 96 days)

### Aggregate measures: total household activity and time spent outside

For the household analyses, we used two main aggregate measures: total household activity and time spent outside. The mean household activity was calculated based on the sum of daily activity across the different house sensors. Sensor total activity was combined from all sensors (PIR, door and appliances) and resampled by household into either daily (from midnight to midnight) or 6 hourly periods (Figs. 2 and 3).

A second analysis focused on the changes in entryway events and time spent outside. The daily entryway events mean was mean-centred to show the relative changes in door activity across the different periods. The time spent outside measure was identified by the combination of: (a) entryway activity [two doors open > close states] for either the front or back door; and (b) the absence of PIR activity within the home until the next entryway event [open to close state]. Time spent outside is then calculated as the time difference between states. Time spent outside was then thresholded by removing any events that lasted less than 3 min and greater than 9 h. This eliminated very short events likely to relate to sensor noise and also events where participants did not return to the home overnight. A standardised measure of mean time spent outside was then calculated to allow comparison across the subjects.

### Statistics

#### Linear mixed-effects modelling of household activity

Linear mixed-effects (LME) multilevel modelling, using the R in Python (‘rpy2’) lme4/lmerTest package^18^, was used to test the relationship between home activity and pandemic phases while considering the individual heterogeneity of the households. Fixed effects of pandemic periods and home occupancy were modelled alongside random effects related to heterogeneity across the individual households. Unless otherwise stated, we use standard significance reporting (i.e.: *p* < 0.0001***, *p* < 0.001**, *p* < 0.05*, *p* < 0.1).

### Reporting summary

Further information on research design is available in the Nature Research Reporting Summary linked to this article.

## Supplementary information

Supplementary information file

Reporting Summary

## Data Availability

The data that support the findings of this study are available from the corresponding author upon reasonable request.

## Code availability

The code used in this study will be made available by the corresponding author upon reasonable request.
