# Supplementary material for: The effect of COVID-19 on the home behaviours of people affected by dementia
Source: NPJ Digit Med. 2022 Oct 17;5:154. doi: 10.1038/s41746-022-00697-4 (PMC9575641; doi:10.1038/s41746-022-00697-4)
Supplement: Supplementary file 1 — Supplementary information file [file 41746_2022_697_MOESM1_ESM.pdf]

Supplementary Information for

"The effect of COVID-19 on the home behaviours of people affected by dementia"

Type of dementia for the participants in the study:

	gender	occupancy	diagnosis	n
0	Female	multiple	Alzheimer's Disease	6
1	Female	multiple	Dementia in Parkinson's	1
2	Female	multiple	Fronto-temporal Dementia	1
3	Female	single	Alzheimer's Disease	5
4	Female	single	Dementia in Parkinson's	1
5	Male	multiple	Alzheimer's Disease	8
6	Male	multiple	Dementia in Parkinson's	3
7	Male	multiple	Fronto-temporal Dementia	1
8	Male	multiple	Vascular Dementia	1
9	Male	single	Alzheimer's Disease	4

Supplementary Table 1: Number of participants according to sex, house occupancy and diagnosis

Inclusion/Exclusion criteria for participants joining the on-going CR&T study:

Person living with dementia/ MCI	
**Inclusion**	**Exclusion**
• Diagnosis of dementia (any type) or mild cognitive impairment by specialist assessment<br>• 50 years and older at baseline<br>• Living in the community<br>• Have a study partner<br>• Sufficient functional English to allow completion of assessments<br>• If lacking in capacity, must have a personal consultee representative	• People with unstable mental state, including severe depression, severe psychosis, agitation, and anxiety who have had medication changed over the last 4 weeks<br>• Severe sensory impairment<br>• Active suicidal ideation<br>• People who require regular elective hospital admission for physical health monitoring<br>• People who are receiving treatment for terminal illness

Study Partner	
**Inclusion**	**Exclusion**
• Willing and able to provide informed consent<br>• Has known the person living with dementia for at least 6 months and is able to attend research assessments<br>• Sufficient functional English to allow completion of the assessments<br>• Aged 18 or over	• Unable to communicate verbally<br>• Unable to provide written informed consent

Supplementary Table 2: Inclusion/exclusion criteria for the participants

Supplementary Note 1: UK Dementia Research Institute (UK DRI) Care Research & Technology (CR&T) Consortium Members and Affiliations

Leadership and Management Infrastructure:

Centre Director: Professor David Sharp[1,3]
Co-Director: Professor Payam Barnaghi[1,3]
Centre Manager: Danielle Wilson[1]
Health and Social Care Lead: Sarah Daniels[1,3]
Project Managers: Mara Golemme[1] and Zaynab Ismail[1]
Group Leaders: Professor David Sharp[1,3], Professor Payam Barnaghi[1,3], Professor Paul Freemont[1,4], Dr Ravi Vaidyanathan[1,5], Professor Tim Constandinou[1,6]
Professor Derk-Jan Dijk[1,7]

Groups:

Behaviour and Cognition led by Prof David Sharp[1,3]

Michael David MD[1,3]
Martina Del Giovane[1,3]
Neil Graham MD PhD[1,3]
Naomi Hassim[1,3]
Magdalena Kolanko MD[1,3]
Helen Lai[1,3]
Lucia Li MD[1,3]
Paresh Malhotra MD PhD[1,3]
Emma Jane Mallas PhD[1,3]
Greg Scott MD[1,3]
Alina-Irina Serban[1,2]
Eyal Soreq PhD[1,3]
Tong Wu PhD[1,3]

Biosensor Hardware led by Prof Timothy Constandinou[1,6]

Alan Bannon PhD[1,6]
Shlomi Haar PhD[1,6]
Charalambos Hadjipanayi[1,6]
Ghena Hammour[1,6]
Bryan Hsieh[1,6]
Adrien Rapeaux PhD[1,6]

Robotics and AI interfaces led by Dr Ravi Vaidyanathan[1,5]

Maria Lima[1,5]
Maitreyee Wairagkar PhD[1,5]

Machine intelligence led by Professor Payam Barnaghi[1,3]

Nan Fletcher-Lloyd[1,3]
Hamed Haddadi PhD[1,3]
Valentinas Janeiko[1,3]
Anna Joffe[1,3]
Samaneh Kouchaki PhD[1,3]
Viktor Levine[1,3]
Honglin Li[1,3]
Amer Marzuki[1,3]
Francesca Palermo[1,3]
Mark Woodbridge[1,3]
Yuchen Zhao PhD[1,3]
Alexander Capstick[1,3]
Severin Skillman[1,3]

Point of care Diagnostics led by Professor Paul Freemont[1,4]

Loren Cameron PhD[1,4]
Michael Crone PhD[1,4]
Kirsten Jensen PhD[1,4]
Martin Tran[1,4]
Thomas Adam[1,4]

Sleep and Circadian led by Professor Derk Jan Dijk[1,7]

Anne Skeldon, PhD[1,7]
Kevin Wells, PhD[1,7]
Ullrich Bartsch PhD[1,7]
Ciro Della Monica PhD[1,7]
Kiran GR Kumar PhD[1,7]
Damion Lambert[1,7]
Sara Mohammadi Mahvash PhD[1,7]
Thalia Rodriguez Garcia PhD[1,7]
Vikki Revell PhD[1,7]
Giuseppe Atzori[1,7]
Lucinda Grainger[1,7]
Hana Hassanin MD[1,7]
James Woolley[1,7]
Iris Wood-Campar[1,7]
Janetta Rexha[1,7]

Human Centred Design led by Matthew Harrison[1,8]

Sophie Horrocks[1,8]
Lenny Naar[1,8]
Brian Quan[1,8]

Site Investigators and Key Personnel:

Surrey and Borders Partnership NHS Foundation Trust (Site and Sponsor)

Chief Investigator: Professor Ramin Nilforooshan
Research and Development Managers: Jessica True, Olga Balazikova
Research Co-ordinator: Emily Beale
Clinical Monitoring Team: Vaiva Zarombaite, Lucy Copps, Olivia Knight, Gaganpreet Bangar, Sumit Dey, Chelsea Mukonda, Jessica Hine, Luke Mallon

Brook Green Medical Centre / Hammersmith and Fulham Site

Principal Investigator: Dr David Wingfield
Research Nurse / Paramedic: Claire Norman
Clinical Studies Officers/Research Technicians: Anesha Patel, Ruby Lyall, Sanara Raza
Research Therapists: Naomi Hassim, Pippa Kirby
LBHF Support: Assistive Technology: John Patterson, Business Development; Mike Law,
Social Services OT: Andy Kenny.

1) UK Dementia Research Institute, Care Research and Technology Centre, London, UK
2) Dyson School of Design Engineering, Imperial College London, London, UK
3) Department of Brain Sciences, Imperial College London, London, UK
4) Department of Infectious Disease, Imperial College London, London, UK
5) Department of Mechanical Engineering, Imperial College London, London, UK
6) Department of Electrical and Electronic Engineering, Imperial College London, London, UK
7) Surrey Sleep Research Centre, Faculty of Health and Medical Sciences, University of Surrey, Guildford, UK
8) HELIX Centre, Imperial College London and Royal College of Art, London, UK
