# Supplementary material for: The effect of COVID-19 on the home behaviours of people affected by dementia
Source: NPJ Digit Med. 2022 Oct 17;5:154. doi: 10.1038/s41746-022-00697-4 (PMC9575641; doi:10.1038/s41746-022-00697-4)
Supplement: Supplementary file 2 — Reporting Summary [file 41746_2022_697_MOESM2_ESM.pdf]

nature portfolio

Corresponding author(s): David Sharp
Last updated by author(s): Sep 12, 2022

# Reporting Summary

Nature Portfolio wishes to improve the reproducibility of the work that we publish. This form provides structure for consistency and transparency in reporting. For further information on Nature Portfolio policies, see our Editorial Policies and the Editorial Policy Checklist.

## Statistics

For all statistical analyses, confirm that the following items are present in the figure legend, table legend, main text, or Methods section.

| n/a | Confirmed | |
|---|---|---|
| ☐ | ☒ | The exact sample size ($n$) for each experimental group/condition, given as a discrete number and unit of measurement |
| ☒ | ☐ | A statement on whether measurements were taken from distinct samples or whether the same sample was measured repeatedly |
| ☐ | ☒ | The statistical test(s) used AND whether they are one- or two-sided<br>*Only common tests should be described solely by name; describe more complex techniques in the Methods section.* |
| ☐ | ☒ | A description of all covariates tested |
| ☐ | ☒ | A description of any assumptions or corrections, such as tests of normality and adjustment for multiple comparisons |
| ☐ | ☒ | A full description of the statistical parameters including central tendency (e.g. means) or other basic estimates (e.g. regression coefficient) AND variation (e.g. standard deviation) or associated estimates of uncertainty (e.g. confidence intervals) |
| ☐ | ☒ | For null hypothesis testing, the test statistic (e.g. $F$, $t$, $r$) with confidence intervals, effect sizes, degrees of freedom and $P$ value noted<br>*Give P values as exact values whenever suitable.* |
| ☒ | ☐ | For Bayesian analysis, information on the choice of priors and Markov chain Monte Carlo settings |
| ☒ | ☐ | For hierarchical and complex designs, identification of the appropriate level for tests and full reporting of outcomes |
| ☒ | ☐ | Estimates of effect sizes (e.g. Cohen's $d$, Pearson's $r$), indicating how they were calculated |

*Our web collection on statistics for biologists contains articles on many of the points above.*

## Software and code

Policy information about availability of computer code

**Data collection**

Data used in this study is collected as part of an on-going study conducted by UK Dementia Research Institute, Care Research and Technology Centre. The data can be accessed using the DCARTE (UK-DRI CAre Research & TEchnology) software developed within the centre. The version of the software used in the COVID-19 study was 0.0.1.

**Data analysis**

Mean household activity was calculated based on the sum of daily activity across the different house sensors. Sensor total activity was combined from all sensors (PIR, door and appliances) and resampled by household into either daily (from midnight to midnight) or six hourly periods.

The time spent outside measure was identified by the combination of: (a) entryway activity [two door open>close states] for either the front or back door; and (b) the absence of PIR activity within the home until the next entryway event [open to close state]. Time spent outside is then calculated as the time difference between states.

Linear mixed-effects (LME) multilevel modelling, using the R in Python (‘rpy2’) lme4/lmerTest package, was used to test the relationship between home activity and pandemic phases while considering the individual heterogeneity of the households. Fixed effects of pandemic periods and home occupancy were modelled alongside random effects related to heterogeneity across the individual households.

For manuscripts utilizing custom algorithms or software that are central to the research but not yet described in published literature, software must be made available to editors and reviewers. We strongly encourage code deposition in a community repository (e.g. GitHub). See the Nature Portfolio guidelines for submitting code & software for further information.

## Data

Policy information about availability of data

All manuscripts must include a data availability statement. This statement should provide the following information, where applicable:

- Accession codes, unique identifiers, or web links for publicly available datasets
- A description of any restrictions on data availability
- For clinical datasets or third party data, please ensure that the statement adheres to our policy

Data will be made available upon reasonable request.

## Human research participants

Policy information about studies involving human research participants and Sex and Gender in Research.

| | |
|---|---|
| Reporting on sex and gender | All the participants identify by sex. This information is collected when the participants join the on-going study conducted by the UK Dementia Research Institute, Care Research and Technology Centre. In the COVID-19 study there are 18 male participants and 13 female participants. |
| Population characteristics | Individuals were living alone in 10 households (4 male and 6 female). The other 21 households had more than one occupant (14 male and 7 female). All participants had a diagnosis of dementia or mild cognitive impairment and were part of an on-going study conducted by UK Dementia Research Institute, Care Research and Technology Centre. |
| Recruitment | Participants had a diagnosis of dementia or mild cognitive impairment, were more than 50 years of age (age range 75 to 93 years old), were living in the community and had a study partner who either lived with them or who were involved in their care. |
| Ethics oversight | Surrey Borders Research Ethics Committee |

Note that full information on the approval of the study protocol must also be provided in the manuscript.

# Field-specific reporting

Please select the one below that is the best fit for your research. If you are not sure, read the appropriate sections before making your selection.

☐ Life sciences ☒ Behavioural & social sciences ☐ Ecological, evolutionary & environmental sciences

For a reference copy of the document with all sections, see nature.com/documents/nr-reporting-summary-flat.pdf

# Behavioural & social sciences study design

All studies must disclose on these points even when the disclosure is negative.

| | |
|---|---|
| Study description | This study is a quantitative analysis of activity levels from the homes of PLwD through the COVID-19 pandemic. Sensor data from the cohort of households was collected before and during the UK COVID-19 lockdowns and was used for continuous assessment of the daily activities. We investigate overall home activity levels and the duration of time spent outside. |
| Research sample | Motion sensor data was collected during the COVID-19 pandemic from 31 households who formed part of an on-going study of dementia conducted by the UK Dementia Research Institute (DRI), Care Research & Technology Centre. Data was continuously collected across a 16-month period inclusive of the three UK national lockdowns. Participants had a diagnosis of dementia or mild cognitive impairment, were more than 50 years of age (age range 75 to 93 years old), were living in the community and had a study partner who either lived with them or who were involved in their care. The study was ethically approved by the Surrey Borders Research Ethics Committee and all participants provided written informed consent. Individuals were living alone in 10 households (4 male and 6 female). The other 21 households had more than one occupant (14 male and 7 female).<br><br>This study uses data collected as part of a larger study which started in April 2019, with participants living with dementia recruited steadily since then. This study is conducted by the UK Dementia Research Institute and it investigates the use of technology to support people living with dementia with the main aim to develop new ways to identify health problems, such as falls and infections that lead to hospital admissions. This dataset comprises of movement data, door data and appliance data. |
| Sampling strategy | No formal sample size calculation was performed for this study because we made use of data collection in an on-going cohort study being conducted by the UK Dementia Research Institute. This is investigating the use of technology to support people living with dementia and was not conceived as a ‘COVID’ study. |
| Data collection | The households in this study had a range of unobtrusive Internet of Things (IoT) sensor technologies that were deployed in the home by Howz. The analysis reported uses anonymised binary data collected by Develco sensors (https://www.develcoproducts.com/) |

| | |
|---|---|
| | from the households. Passive infrared sensors (Develco Motion Sensor Mini) were installed in the bedroom, the lounge/living room, the kitchen, the bathroom and the hallway. Sensor placement varies according to the house layout, such that the sensors are placed within the most active locations within the house. Door sensors (Develco Window Sensor) were placed on the front door, the back door and the fridge door, and they collect information about a door being open or close. Two smart plugs (Develco Smart Plug Mini) were placed on kitchen appliances such as the kettle and toaster or microwave, allowing the collection of data about the usage of these appliances. |
| Timing | This study is part of an on-going study that started in April 2019. The analysis timeline starts on 1st December 2019 and ends on 12th April 2021. |
| Data exclusions | For the time spent outside analysis, all data below 3 minutes or higher than 9 hours. This eliminated very short events likely to relate to sensor noise and also events where participants did not return to the home overnight. |
| Non-participation | No participants dropped out/ declined participation during the COVID study. |
| Randomization | Participants were not allocated into experimental groups. |

# Reporting for specific materials, systems and methods

We require information from authors about some types of materials, experimental systems and methods used in many studies. Here, indicate whether each material, system or method listed is relevant to your study. If you are not sure if a list item applies to your research, read the appropriate section before selecting a response.

## Materials & experimental systems

| n/a | Involved in the study | |
|---|---|---|
| ☒ | ☐ | Antibodies |
| ☒ | ☐ | Eukaryotic cell lines |
| ☒ | ☐ | Palaeontology and archaeology |
| ☒ | ☐ | Animals and other organisms |
| ☒ | ☐ | Clinical data |
| ☒ | ☐ | Dual use research of concern |

## Methods

| n/a | Involved in the study | |
|---|---|---|
| ☒ | ☐ | ChIP-seq |
| ☒ | ☐ | Flow cytometry |
| ☒ | ☐ | MRI-based neuroimaging |
